# Clinical implementation of polygenic risk scores

**DOI:** 10.1038/s41431-025-01931-9

**Published:** 2025-09-29

**Authors:** E. Roberts, N. Flaum, D. G. Evans

**Affiliations:** 1 Division of Cancer Sciences, School of Medical Sciences, Faculty of Biology, Medicine and Health, The University of Manchester, Manchester, UK; 2 Manchester Breast Centre, The Christie, Manchester, UK; 3 Manchester Centre for Genomic Medicine, St Mary’s Hospital, Manchester Academic Health Centre (MAHSC), Division of Evolution, Infection and Genomic Sciences, University of Manchester, Manchester, UK; 4 Prevent Breast Cancer Prevention Centre, University Hospital of South Manchester NHS Trust, Wythenshawe, Manchester, UK; 5 Manchester Centre for Genomic Medicine, St Mary’s Hospital, Central Manchester University Hospitals NHS Foundation Trust, Manchester, UK

**Keywords:** Genetics, Breast cancer

Risk of a disease is not binary but a continuum. The difficulty in ascertaining an individual’s risk is further complicated as it is almost always multifactorial and will evolve over time. While stratified screening exists in certain diseases for higher risk individuals, this is frequently based on outdated and simplistic criteria. Accurate risk estimation is essential for medical professionals to give fully informed advice about risk reduction strategies, which range from tailored surveillance with imaging to chemoprevention or risk-reduction surgery.

The majority of genetic risk of diseases are not explained by monogenic pathogenic variants (PVs). Genome Wide Association Studies (GWAS) have identified multiple common but lower penetrance susceptibility Single Nucleotide Polymorphisms (SNPs), which in combination in a Polygenic Risk Score (PRS) explain a large portion of the unknown inherited component [1]. PRSs are currently being incorporated in large-scale clinical trials, including Our Future Health (www.ourfuturehealth.org.uk) which is recruiting five million UK adults. For breast cancer, PRSs have been widely investigated in trials including Predicting Risk of Cancer At Screening (PROCAS), My Personal Breast Screening (MyPeBS) and Women Informed to Screen Depending on Measures of Risk (WISDOM) but are currently also available in commercial direct-to-consumer testing. PRSs are entering the mainstream of medicine and are viewed with both hope and scepticism even amongst the genetics community.

**Fig. 1:**
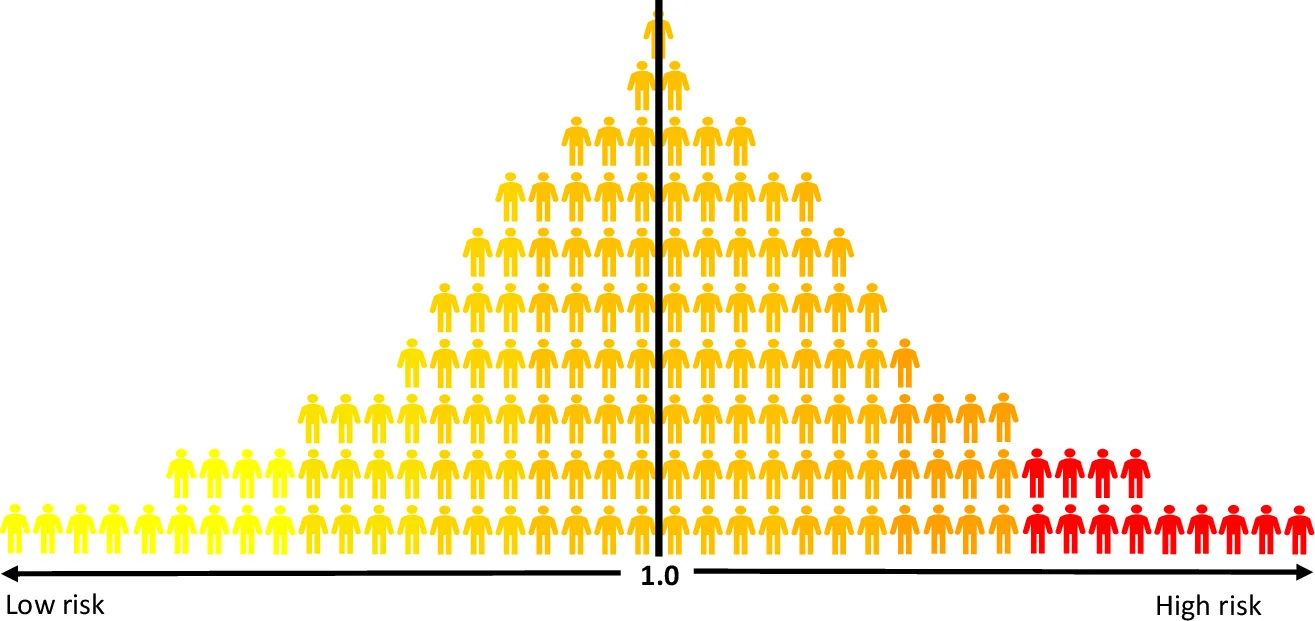
PRS normally distribute across the population [1].

PRSs are validated in specialties including cardiology, psychiatry, rheumatology, endocrinology and oncology. In some cases they have better performance in risk prediction and diagnosis than ‘traditional’ blood tests or imaging, for example PRSs have better discriminatory capacity for ankylosing spondylitis than C-reactive protein (CRP), sacroiliac Magnetic Resonance Imaging (MRI), or *HLA-B27* status [2], and for breast cancer (BC) the SNP313 PRS has an estimated 35% contribution to familial BC relative risk, with >50% of people having a risk 1.5-fold higher or lower than population average (Fig. 1) [3, 4]. The Polygenic Score Catalog (www.pgscatalog.org) is a regularly updated repository of PRSs developed for a variety of diseases and metrics. Indeed, on a population basis PRSs have far more of an effect on risk stratification of breast cancer on a population basis than monogenic moderate/high risk genes with only 1.7% who carry pathogenic variants having a meaningful change in risk [4]. Additionally, by considering risk equivalently across both PRSs and PVs, it could be argued that an individual with a high PRS which would place them at similar cancer risk as a PV carrier should be entitled to comparable enhanced early detection and prevention techniques.

Numerous studies have demonstrated that risk prediction models (RPMs) are optimised by the combination of PRS with clinical risk factors. For BC, one study reported an Area Under the Curve (AUC) of 0.677 when including classic risk factors, breast density, SNP313 and a gene panel, compared with 0.536 for classic risk factors alone [4]. PRSs can also risk stratify more accurately than single pathogenic variants (PVs) in a high or moderate risk genes. The Cancer Risk Estimates Related to Susceptibility (CARRIERS) consortium found that a breast cancer PRS had the ability to stratify >30% of *CHEK2* and ∼50% of *ATM* PV carriers as <20% lifetime risk [5]. Breast cancer PRSs have already been clinically implemented, having been incorporated in the CanRisk (BOADICEA) and Tyrer-Cuzick RPMs, of which the former already has CE marking within the European Economic Area (EEA).

A crucial question is acceptability of any test. PROCAS/BC-Predict, MyPeBS and WISDOM have been investigating the acceptability of BC RPMs which incorporate PRS and their clinical implementation. BC-Predict specifically investigated the psychological impact of risk stratification including PRSs within the PROCAS cohort and found cancer worry scores were no different comparing women in BC-Predict and those on the standard NHS Breast Screening Programme [6]. They also concluded that the addition of a PRS is feasible for risk assessment, can be delivered in reasonable time and could be cost effective for the NHS. A specific micro-costing study has estimated that incorporating a PRS in risk stratification would cost only £78 [7], with any raw genetic information able to generate PRSs for many diseases.

Whilst oncological PRSs typically only predict risk of disease development, PRSs in other disease areas can aid diagnostic refinement and predict disease progression and/or recurrence. For diabetes, a 30-SNP PRS showed high discriminatory ability for differentiation of Type 1 and Type 2 Diabetes (T1D and T2D) with an AUC of 0.88 alone or of 0.96 with other clinical risk factors) [8]. A T2D PRS has also been shown to predict disease progression, namely the transition from normal glucose tolerance to prediabetes and from prediabetes to T2D disease state. The individuals in the most severe diabetes subgroup also had significantly high T2D PRSs and were clinically characterised by resistance to insulin and β-cell dysfunction. Evidence has also shown that cardiovascular PRSs have improved risk discrimination for future adverse/recurrent cardiovascular events including myocardial infarction and ischemic stroke, among those with pre-existing cardiovascular diseases [9].

**Fig. 2:**
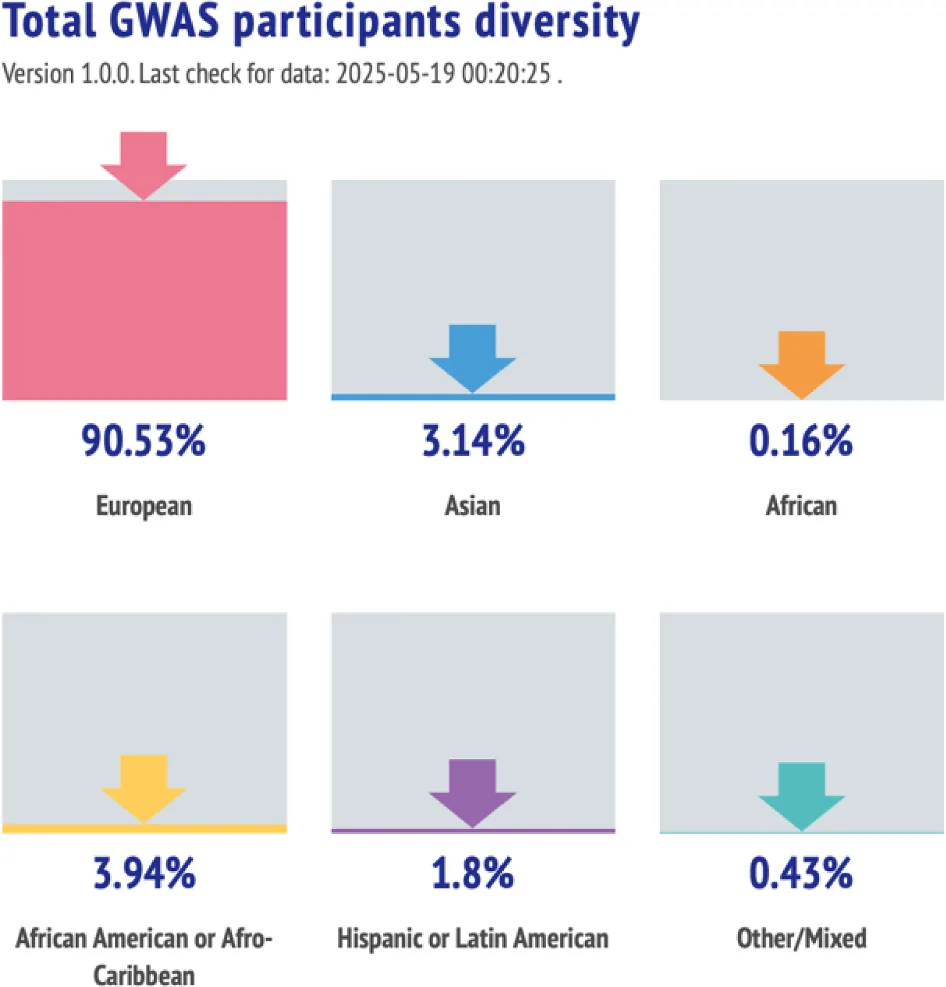
GWAS participants diversity by May 2025 across all disease types (www.gwasdiversitymonitor.com).

There are recognised limitations of PRS in clinical applicability. Currently, the main limitation is the lack of diversity in participants to the initial GWASs from which the PRSs were built, and therefore relevance to non-Caucasian populations. 91% of all GWAS data is generated from those of European descent. The GWAS diversity monitor, a dashboard assessing GWAS ancestral diversity in real-time, showed only ∼4% of GWAS in all diseases carried out in people of African ancestry, ∼3% in Asians (mostly East Asian) and ∼2% in Hispanic (Fig. 2). Diverse representation in GWAS is vital to identify disease-associated SNPs in multiple ancestral groups, determine an accurate risk score and to assign each SNP’s risk in the correction direction. Currently, it is known that PRSs overestimate risk in populations not of European origin with the greatest overprediction in African populations, although work is ongoing to improve the use of PRSs in diverse populations. For example, a simple correction has been calculated for the use of PRS in BC risk prediction in Ashkenazi people [10]. These corrections could allow accurate PRS calculation and should be explored further in various ancestries. Multi Ancestry PRS (MA-PRS) are relatively novel and show promise. Generally, they include both disease-associated and ancestry-informative SNPs although MA-PRS methodology currently has limitations with minimal acknowledgment of complex genetic architecture within populations.

Another consideration that will face increased scrutiny and standardisation as PRSs are increasingly used in clinical practice is that there is no current standardised or regulated method of PRS development or validation, and the computational Quality Control (QC) steps and protocol used for each PRS generated are not always clearly described or understood by clinicians who are required to implement them. Additionally, using different PRSs for the same disease can lead to discordance regarding those classified at highest risk, with large differences in individual risk estimation. This could lead to patients being offered different medical advice/interventions, depending on the PRS used, and this uncertainty is a vital topic to better understand and resolve. Good risk communication is vital, such that the clinical question for each PRS generated is explained to individuals, whether that is disease risk prediction, progression or recurrence, among others. It is also important that absolute risk is discussed with any patient rather than relative risk, to facilitate improved understanding. For example, with UK population risk at 11–12% for BC, a woman with a PRS of 1.5 or a 50% relative risk increase, will actually have an absolute risk increase of only 5–6%.

Clinically, a score that incorporates genetic data from PVs and PRS, in combination with clinical factors, can provide the most accurate personalised risk estimate for an individual across multiple disease areas. PRSs have become an essential part of personalised risk prediction. Policy decision-makers from the UK National Screening Committee (NSC) are investigating risk-stratified screening, but acknowledge its implementation requires further work including improvements in digital infrastructure and considerations about which specific risk assessment tool to use, among others. Ultimately, population screening could be greatly improved across many diseases with the use of PRSs. With PRSs already transitioning into clinic in some diseases, it is vital that research and clinic work together with full transparency to implement appropriate structures to maximise PRS effectiveness for all patients and public.
